# An analysis of Public Health England’s vaccination data for care home workers immediately preceding a ‘Roadmap out of COVID-19 Lockdown’

**DOI:** 10.3399/BJGPO.2021.0152

**Published:** 2022-01-12

**Authors:** Paula Swift, Lisa Bunn

**Affiliations:** 1 Mount Gould Hospital, Livewell Southwest, Plymouth, UK; 2 Faculty of Health, University of Plymouth, Plymouth, UK

**Keywords:** Community care, Care home workers, COVID vaccination, General practice, Primary healthcare

## Background

On March 8th 2021, a *Roadmap out of C* *OVID* *-19 Lockdown* was activated, informed by Public Health England’s (PHE) surveillance and open access reporting of infection and vaccination rates.^1^ The PHE open dataset used for the *Roadmap* was published on 25th February 2021, and included total number of first and second vaccinations in England and vaccination count of all older adult care home workers.^2^

Children returned to school, based on the government’s impression that vaccination rates for people in the priority levels were sufficient.^1^

Acknowledging the vulnerability of older adult care home residents to COVID-19, the Scientific Advisory Group (SAGE) recommended an 80% vaccine uptake in older adult care home workers. While the rationale behind the 80% remains largely unreported, it was felt to be a critical factor in achieving the aims of the vaccination programme,^3^ namely: to open up society; to prevent healthcare from becoming overwhelmed; and to reduce severe illness.^4^ By 25th February, all older adult care home workers had been offered a first vaccination^5^ and a decision had been made to proceed with the *Roadmap*. An assumption could therefore be made that >80% of older adult care home workers in all regions, regardless of the infection rate, had at this point been vaccinated with at least a single vaccination. It is, however, plausible that care home workers in regions of high infection rates, particularly in the North West and Midlands, may have a vaccination rate in excess of 80%. In this commentary, we analyse the 25th February data from PHE to test these theories and explore the following questions (Q):

Q1. What was the percentage first vaccine uptake by older adult care home staff across England immediately ahead of the **Roadmap out of C* *OVID* *-19 Lockdown**?

Q2. Did this consistently achieve 80% in all regions of England?

Q3. Was there a difference in vaccine uptake across regions in England?

### Analysis

Data from the 25th February 2021 PHE dataset^6^ were imported from an Excel workbook (Microsoft Office, 2019) to SPSS Statistics (version 25) and coded for analysis. Errors were checked and none detected following import. A new variable, the percentage first vaccine uptake by care home workers in England, was calculated (Equation 1).

Equation 1: *Calculating a percentage vaccination uptake measure*

Care home staff who had tested positive for COVID-19 (within 28 days) were excluded to ensure that only staff eligible for the vaccine were included in the calculation. The percentage uptake of first vaccination was calculated by region and visualised descriptively using cross-tabulation and bar charting methods.

The following null hypothesis was tested to explore whether region was associated with vaccination uptake (research Q3):

Binary data per region (vaccination received: yes = 1; no = 2), enabled 2 × 2

contingency tables to be drawn to test the null hypothesis. Tests of association against a ‘benchmark’ region with the vaccination uptake closest to the 80% SAGE recommendation were then explored.

Non-parametric inferential statistics were used to identify any statistical association between vaccination uptake in each region against the benchmark, namely the

Pearson Χ^2^ test and Yate’s Correction for Continuity, compensating for the over-estimate of Χ^2^.^7^ Effect size was measured using the Phi coefficient and assessed against Cohen’s criteria of effect size.^8^ Since Phi coefficient can be affected by unequal response frequencies,^9^ measure of effect size for any significant association reported *P*<0.05 were presented conservatively as univariate odds ratios.^10^

### Findings

Approximately 444 000 care home workers in England were identified as eligible for COVID-19 first vaccination. The uptake of first vaccination was 72.6% (*n* = 322 534), which is 7.4% (*n* = 32 871) short of the 80% care home worker target value set by SAGE^2^ (Table 1).

**Table 1.** Summary COVID-19 vaccination figures for care home workers by region in England immediately preceding the *Roadmap out of* *COVID* *-19 Lockdown* implementation

Region in England	Eligible population, *n*	Vaccinated, *n*	Unvaccinated, *n*	Vaccination uptake in eligible population, %	Percentage required to achieve 80%, %
North West	58 871	41 754	17 117	70.1	9.7
North East and Yorkshire	72 627	56 012	16 615	77.1	2.9
Midlands	89 041	66 857	22 184	75.1	4.9
London	34 001	18 733	15 268	55.1	24.9
East	52 358	36 798	15 560	70.3	9.7
South East	79 965	59 317	20 648	74.2	5.8
South West	57,337	43,063	14,274	75.1	4.9
Total	444,200	322,534	121,666	72.6	7.4

**Figure 1.**
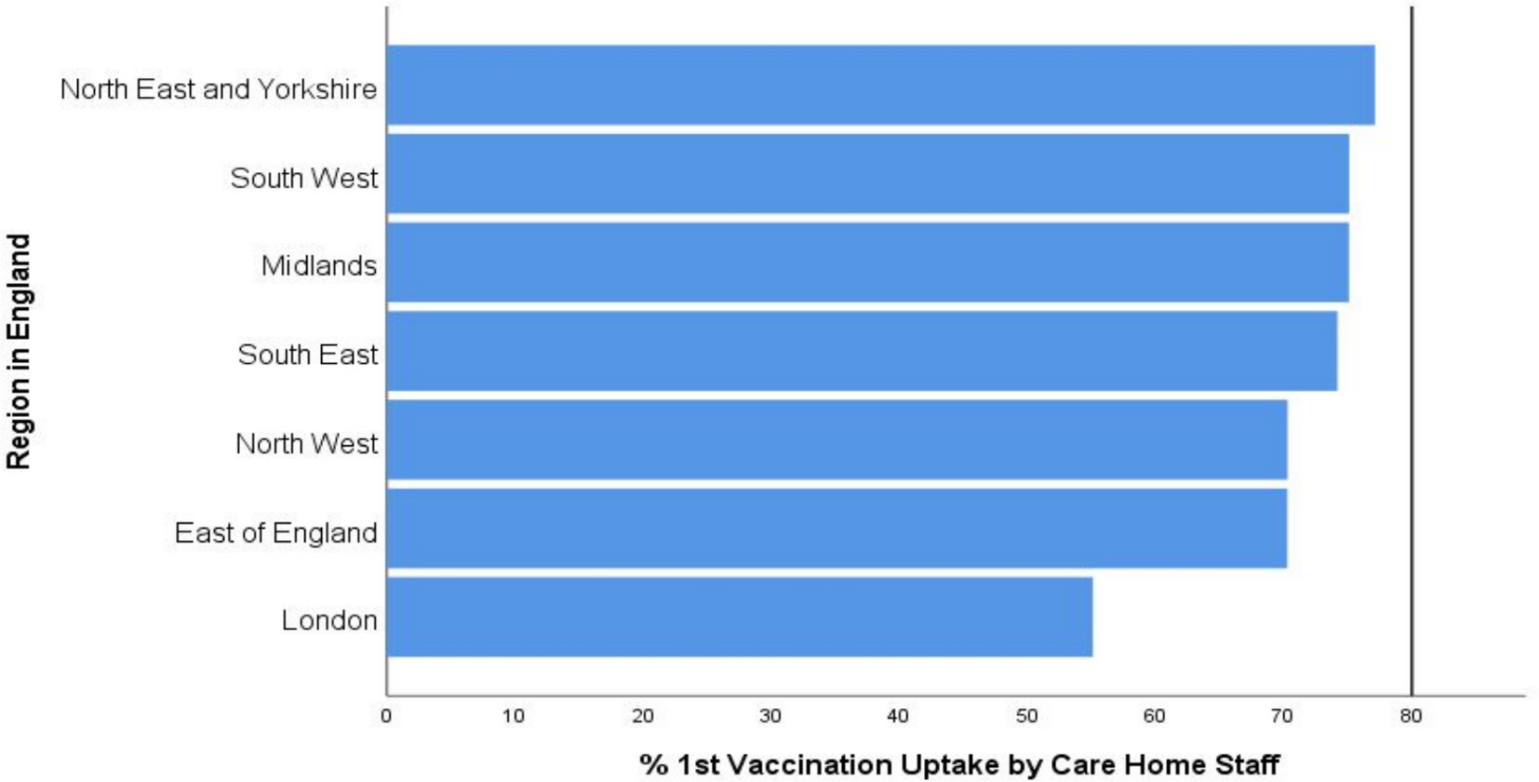
Percentage first vaccination uptake in care home staff by region in England immediately preceding the implementation of *Roadmap out of* *COVID* *-19 Lockdown*. Recommended vaccine uptake rate (80%) denoted by reference line^2^

Across the seven main regions of England, uptake rate was highest in the North East and Yorkshire (77.1%, *n* = 56 012) and lowest in London (55.1%, *n* = 18 733). While there was a reasonable consistency between 6 of the regions, with vaccination uptake ranging from 70.1–77.1%, London was 14.9% below this lower range. None of the regions achieved the recommended 80% vaccination uptake (Figure 1). Three regions were within 5% of this target, including the North East (-2.9%), South West (-4.9%), and Midlands (-4.9%).

The North East was used as the benchmark region against which to test the research Q3 null hypothesis. Table 2 outlines the Χ^2^ analysis of difference between the North East and all other regions, as well as effect size by Phi coefficient and Cohen’s criteria.^8^ The effect size using Phi was greatest between the North East and East regions (–.077), rather than the North East and London, suggesting that the results may be affected by unequal response frequencies, since there were fewer care home workers in some regions.^9^ Univariate odds ratios, considered a more stable measure of effect size,^10^ reported a reduction in the odds of first vaccination uptake across all regions compared to the North East. Individuals were just over one-third as likely to be vaccinated in London compared to the North East (OR 0.36, 95% CI 0.35 to 0.37), while in the South West care home workers were only slightly less likely to be vaccinated compared to those in the North East (OR 0.90, 95% CI 0.87 to 0.92). On balance, these data act to reject the null hypothesis that there was no difference in vaccination uptake across regions of England.

**Table 2.** Vaccination uptake by region using North East England and Yorkshire as a benchmark

Region	Odds ratio	Confidence interval (CI)	*P* value(*X^2^*)	Effect size(Phi)
North West	0.72	0.71 to 0.74	<0.001	–0.71
Midlands	0.89	0.87 to 0.92	<0.001	–0.24
London	0.36	0.35 to 0.37	<0.001	–0.22
East	0.70	0.68 to 0.72	<0.001	–0.77
South East	0.85	0.83 to 0.87	<0.001	–0.34
South West	0.90	0.87 to 0.92	<0.001	–0.24

### Interpretation

This analysis identifies that at the point of activating England’s *Roadmap out of C* *OVID* *-19 Lockdown*, just under three-quarters (72.6%) of older adult care home workers eligible had received a COVID-19 vaccination.

In view of the broad media coverage concerning the vulnerability of care home residents and the negative impact that COVID-19 has had on care homes,^11^ we anticipated that vaccine uptake would have been high, in excess of the SAGE target in regions where the infection rate was highest. None of the regions, however, met the SAGE care home vaccination target of 80%.^2^ While the North East and Yorkshire, known to have a particularly high infection rate, did indeed achieve the highest rate of vaccination uptake, regional variation in uptake was significant (*P*<0.001). The difference in regional uptake continued into May 2021, with 55.2% of care home workers in London having received both doses of COVID-19 vaccination, compared with 73.2% in the North East region.^12^ This analysis of PHE open data supports the importance of exploring factors influencing vaccine uptake, in order to guide future public health strategies in this social care sector.^13^ London and the South East demonstrated a slow initial roll-out across all early groups offered the vaccine, and vaccine hesitancy was found to be high in older adult care home workers in London, particularly among those in BAME [sic] groups,^14^ providing some explanation around the regional differences observed within the data. Examples of hesitancy included mistrust, complacency, a lack of sufficient information, sociodemographic factors, logistical barriers, and ethnicity.^15^ It is important to highlight that the 80% vaccination target set by SAGE^2^ is guidance only. Research focusing on vaccine hesitancy appears to accept the validity of this fundamental guidance, but without an evaluation of the consequences of not following the guidance, or an evaluation of the evidence base that underpins this guidance, the precision of an 80% vaccination rate in epidemic management is difficult to assess, and would itself benefit from further research.

Strategic attempts to increase vaccination uptake among older adult care home workers initially included targeted programmes aimed at ensuring accessibility of vaccines to care home workers and offering communications to increase their knowledge and understanding of the benefits of vaccination.^13^ Vaccination amongst older adult care home workers has been mandated since 11th November 2021.^13^ The evidence base underpinning this decision to effectively increase the vaccination rate from 80% to 100% in the care home sector remains uncertain. Mandated vaccination could explain the recent increase in fully vaccinated staff across all regions to 88.9%, with London still having the lowest uptake at 87.5%.^16^ Of note, while the percentage vaccination rate rose, the percentage eligible is no longer reported and the total number of staff across England fell by 4259 (between 25^th^ February and 28^th^ October 2021), with a loss of 830 from the London region alone.^16^ Without eligibility status or underlying reasons for persistent vaccine hesitancy being determined for the remaining 11.1% of unvaccinated workers, this could lead to large numbers of staff leaving the care home workforce, increasing the staffing crisis within this sector and further impacting patient safety.^17^

It is impossible to now explore whether ‘softer’ measures — such as education and accessibility of vaccines, particularly for specific ethnic groups — could be as effective as mandatory vaccination within England’s older adult care home sector, but the impact of mandatory vaccination across all regions now clearly warrants an evaluation to understand the impact on employment and retention rates.
